# (*Z*)-9-(1,2-Dichloro­vin­yl)-9*H*-carbazole

**DOI:** 10.1107/S1600536813007137

**Published:** 2013-03-20

**Authors:** Miho Ukai, Hideyuki Tabata, Tsunehisa Okuno

**Affiliations:** a Department of Material Science and Chemistry, Wakayama University, Sakaedani, Wakayama, 640-8510, Japan

## Abstract

There are two independent mol­ecules in the asymmetric unit of the title compound, C_14_H_9_Cl_2_N, in which the dihedral angles between the dichloro­vinyl unit (r.m.s. deviations = 0.0003 and 0.0009 Å), and the carbazole ring are 87.77 (3) and 72.90 (3)°.

## Related literature

For the preparation of the title compound, see: Okamoto & Kundu (1970 ▶); Cuniberti *et al.* (1996 ▶). For the structure of 9-vinyl­carbazole, see: Tsutsui *et al.* (1976 ▶); Tian *et al.* (2006 ▶). For the optoelectric properties of vinyl­carbazoles, see: Ye *et al.* (2010 ▶). 
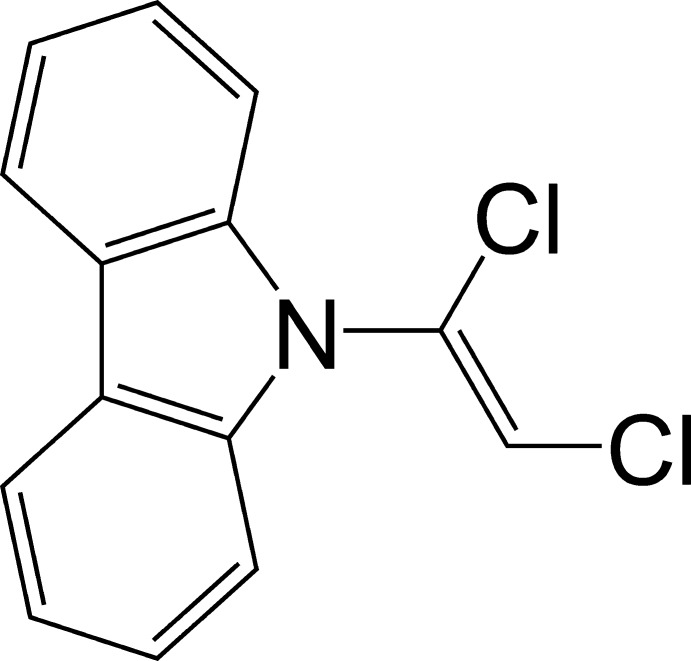


## Experimental

### 

#### Crystal data

C_14_H_9_Cl_2_N
*M* *_r_* = 262.14
Monoclinic, 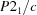

*a* = 6.6552 (7) Å
*b* = 24.872 (3) Å
*c* = 14.8636 (15) Å
β = 93.7529 (16)°
*V* = 2455.1 (5) Å^3^
*Z* = 8
Mo *K*α radiation
μ = 0.50 mm^−1^
*T* = 93 K
0.14 × 0.11 × 0.07 mm

#### Data collection

Rigaku Saturn724+ diffractometer
Absorption correction: numerical (*NUMABS*; Rigaku, 1999 ▶) *T*_min_ = 0.939, *T*_max_ = 0.965
19841 measured reflections
5595 independent reflections
5154 reflections with *F* ^2^ > 2σ(*F* ^2^)
*R*_int_ = 0.024

#### Refinement

*R*[*F* ^2^ > 2σ(*F* ^2^)] = 0.035
*wR*(*F* ^2^) = 0.092
*S* = 1.04
5594 reflections
323 parameters
H-atom parameters constrained
Δρ_max_ = 0.60 e Å^−3^
Δρ_min_ = −0.43 e Å^−3^

### 

Data collection: *CrystalClear* (Rigaku, 2008 ▶); cell refinement: *CrystalClear*; data reduction: *CrystalClear*; program(s) used to solve structure: *SIR92* (Altomare *et al.*, 1994 ▶); program(s) used to refine structure: *SHELXL97* (Sheldrick, 2008 ▶); molecular graphics: *ORTEP-3* forWindows (Farrugia, 2012 ▶); software used to prepare material for publication: *CrystalStructure* (Rigaku, 2010 ▶).

## Supplementary Material

Crystal structure: contains datablock(s) global, I. DOI: 10.1107/S1600536813007137/qm2093sup1.cif

Structure factors: contains datablock(s) I. DOI: 10.1107/S1600536813007137/qm2093Isup2.hkl

Supplementary material file. DOI: 10.1107/S1600536813007137/qm2093Isup3.cml

Additional supplementary materials:  crystallographic information; 3D view; checkCIF report

## Figures and Tables

**Fig. 1.**
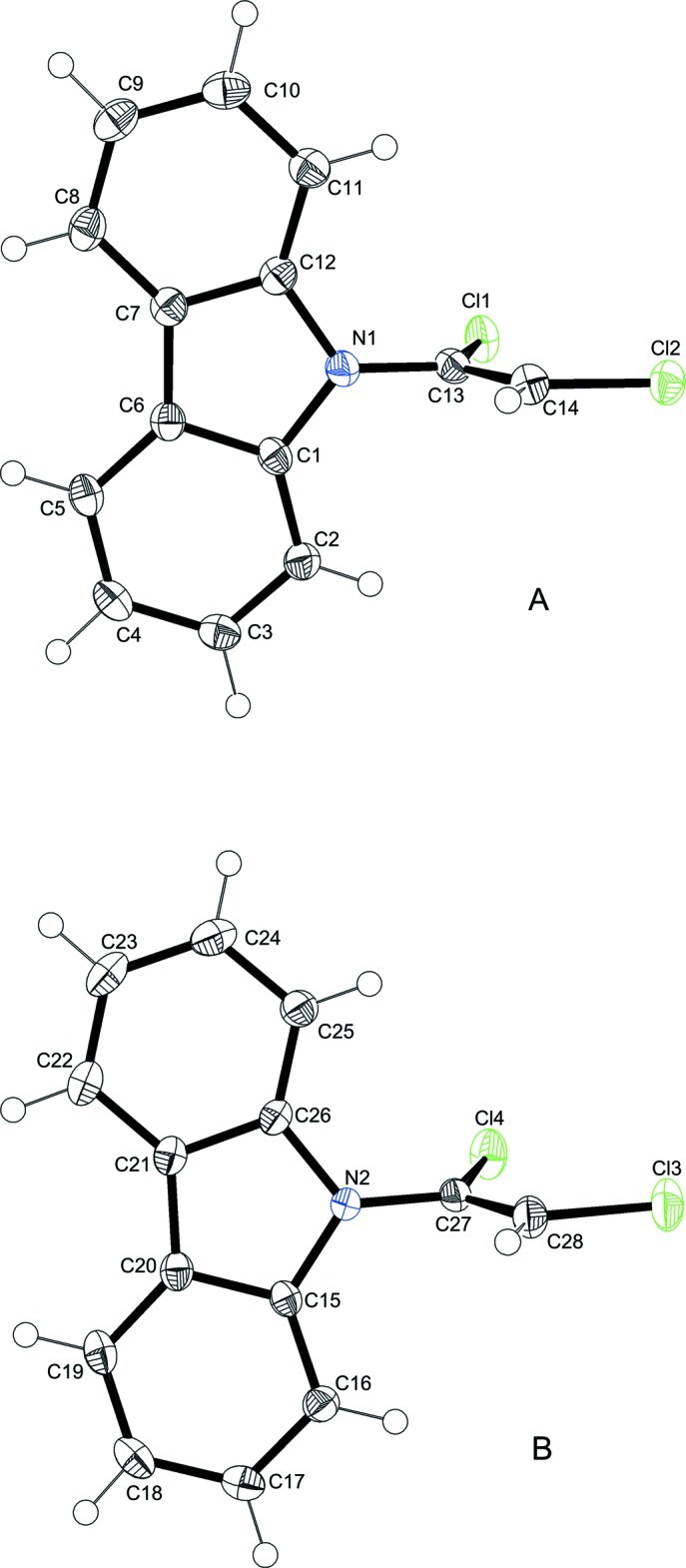
The asymmetric unit of the title compound with atom-numbering scheme. Displacement ellipsoids are drawn at the 50% probability level and H atoms are shown as small spheres.

**Fig. 2.**
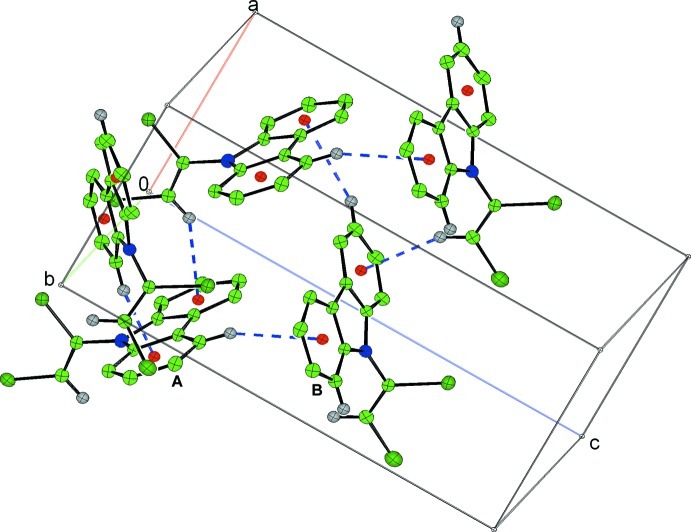
A portion of the lattice showing the C-H···π contacts with H atoms not involved in the C-H···π contacts omitted for clarity.

## supplementary crystallographic information

### Comment

Vinylcarbazoles are interesting as monomers of poly(*N*-vinylcarbazoles) which have been attracted interest from the viewpoint of their optoelectric properties (Ye *et al.*, 2010). The title compound, C_14_H_9_N_1_Cl_2_, is a halogen analogue of vinylcarbazole. In the literature (Okamoto & Kundu, 1970), the compoud is a mixture of *E* and *Z* forms. The crystals, however, were found to have only a *Z* form.

There are two independent molecules (A and B) in the asymmetric unit (Fig. 1). The carbazole rings are planar (N1/C1—C12 plane: r.m.s. deviation = 0.0194 Å, N2/C15—C26 plane: r.m.s. deviation = 0.0377 Å). The dichlorovinyl parts are also planar (Cl1/Cl2/C13/C14 plane: r.m.s. deviation = 0.0009 Å, Cl3/Cl4/C27/C28 plane: r.m.s. deviation = 0.0003 Å). The dihedral angles between the dichlorovinyl part and the carbazole ring are 87.77 (3)° for A and 72.90 (3)° for B, which are larger than those in vinylcarbazole (Tsutsui *et al.*, 1976 and Tian *et al.*, 2006), suggesting steric repulsion of the Cl1 or Cl3 with the hydrogen atoms of the carbazole rings. The inter-molecular contacts are dominated by C-H···π interactions which involve both six-membered rings of both molecules A and B (Fig. 2).

### Experimental

The title compound was prepared according to a published procedure (Cuniberti *et al.*, 1996). The single crystals with sufficient quality for X-ray analysis were grown slowly from the oilish mixture.

### Refinement

The C-bound H atoms were placed at ideal positions and were refined as riding on their parent C atoms. *U*_iso_(H) values of the H atoms were set at 1.2*U*_eq_(parent atom).

### Crystal data

**Table:**

C_14_H_9_Cl_2_N	*F*(000) = 1072.00
*M**_r_* = 262.14	*D*_x_ = 1.418 Mg m^−^^3^
Monoclinic, *P*2_1_/*c*	Mo *K*α radiation, λ = 0.71075 Å
Hall symbol: -P 2ybc	Cell parameters from 9066 reflections
*a* = 6.6552 (7) Å	θ = 1.6–31.3°
*b* = 24.872 (3) Å	µ = 0.50 mm^−^^1^
*c* = 14.8636 (15) Å	*T* = 93 K
β = 93.7529 (16)°	Block, colorless
*V* = 2455.1 (5) Å^3^	0.14 × 0.11 × 0.07 mm
*Z* = 8	

### Data collection

**Table:**

Rigaku Saturn724+ diffractometer	5154 reflections with *F*^2^ > 2σ(*F*^2^)
Detector resolution: 28.445 pixels mm^-1^	*R*_int_ = 0.024
ω scans	θ_max_ = 27.5°
Absorption correction: numerical (*NUMABS*; Rigaku, 1999)	*h* = −8→8
*T*_min_ = 0.939, *T*_max_ = 0.965	*k* = −32→32
19841 measured reflections	*l* = −19→19
5595 independent reflections	

### Refinement

**Table:**

Refinement on *F*^2^	Secondary atom site location: difference Fourier map
*R*[*F*^2^ > 2σ(*F*^2^)] = 0.035	Hydrogen site location: inferred from neighbouring sites
*wR*(*F*^2^) = 0.092	H-atom parameters constrained
*S* = 1.04	*w* = 1/[σ^2^(*F*_o_^2^) + (0.0454*P*)^2^ + 1.5809*P*] where *P* = (*F*_o_^2^ + 2*F*_c_^2^)/3
5594 reflections	(Δ/σ)_max_ < 0.001
323 parameters	Δρ_max_ = 0.60 e Å^−^^3^
0 restraints	Δρ_min_ = −0.43 e Å^−^^3^
Primary atom site location: structure-invariant direct methods	

### Special details

**Table:**

Refinement. Refinement was performed using all reflections except for one with very negative *F*^2^. The weighted *R*-factor (*wR*) and goodness of fit (*S*) are based on *F*^2^. *R*-factor (gt) are based on *F*. The threshold expression of *F*^2^ > 2.0 σ(*F*^2^) is used only for calculating *R*-factor (gt).

### Fractional atomic coordinates and isotropic or equivalent isotropic displacement parameters (Å^2^)

**Table:**

	*x*	*y*	*z*	*U*_iso_*/*U*_eq_	
Cl1	0.36938 (6)	0.115051 (16)	−0.06836 (2)	0.02685 (10)	
Cl2	−0.07609 (5)	0.067708 (15)	−0.04789 (3)	0.02598 (10)	
Cl3	−0.48776 (6)	0.417277 (18)	0.20176 (3)	0.03522 (11)	
Cl4	−0.02371 (6)	0.385892 (18)	0.22034 (3)	0.03179 (11)	
N1	0.38706 (19)	0.12141 (5)	0.11169 (8)	0.0220 (3)	
N2	−0.07119 (18)	0.36020 (5)	0.04603 (8)	0.0187 (3)	
C1	0.4098 (3)	0.17400 (6)	0.14528 (9)	0.0208 (3)	
C2	0.2888 (3)	0.21869 (6)	0.12645 (10)	0.0237 (3)	
C3	0.3395 (3)	0.26575 (6)	0.17308 (10)	0.0255 (3)	
C4	0.5047 (3)	0.26787 (6)	0.23673 (10)	0.0263 (3)	
C5	0.6246 (3)	0.22303 (6)	0.25421 (10)	0.0240 (3)	
C6	0.5777 (3)	0.17551 (6)	0.20749 (9)	0.0209 (3)	
C7	0.6640 (3)	0.12206 (6)	0.21095 (10)	0.0216 (3)	
C8	0.8266 (3)	0.09901 (7)	0.26128 (10)	0.0264 (3)	
C9	0.8666 (3)	0.04476 (7)	0.24977 (11)	0.0284 (4)	
C10	0.7481 (3)	0.01371 (6)	0.18843 (11)	0.0267 (4)	
C11	0.5848 (3)	0.03562 (6)	0.13764 (10)	0.0246 (3)	
C12	0.5451 (3)	0.08984 (6)	0.15036 (10)	0.0213 (3)	
C13	0.2646 (3)	0.10640 (6)	0.03541 (10)	0.0205 (3)	
C14	0.0817 (3)	0.08677 (6)	0.04245 (10)	0.0231 (3)	
C15	−0.0809 (2)	0.30780 (6)	0.01076 (9)	0.0184 (3)	
C16	−0.2113 (3)	0.26628 (6)	0.02964 (10)	0.0220 (3)	
C17	−0.1905 (3)	0.21842 (6)	−0.01740 (10)	0.0243 (3)	
C18	−0.0458 (3)	0.21255 (6)	−0.08086 (10)	0.0260 (3)	
C19	0.0838 (3)	0.25415 (6)	−0.09896 (10)	0.0246 (3)	
C20	0.0660 (3)	0.30276 (6)	−0.05279 (9)	0.0202 (3)	
C21	0.1688 (2)	0.35412 (6)	−0.05645 (10)	0.0210 (3)	
C22	0.3168 (3)	0.37452 (7)	−0.10978 (11)	0.0271 (4)	
C23	0.3715 (3)	0.42804 (7)	−0.10051 (12)	0.0305 (4)	
C24	0.2833 (3)	0.46122 (6)	−0.03795 (12)	0.0282 (4)	
C25	0.1378 (3)	0.44174 (6)	0.01656 (10)	0.0230 (3)	
C26	0.0810 (2)	0.38833 (6)	0.00524 (10)	0.0192 (3)	
C27	−0.1707 (3)	0.37955 (5)	0.11931 (9)	0.0188 (3)	
C28	−0.3629 (3)	0.39276 (6)	0.11315 (11)	0.0246 (3)	
H2	0.1793	0.2175	0.0877	0.0283*	
H3	0.2536	0.2973	0.1612	0.0286*	
H4	0.5326	0.3008	0.2696	0.0249*	
H5	0.7354	0.2243	0.2985	0.0314*	
H8	0.9082	0.1201	0.3026	0.0317*	
H9	0.9758	0.0285	0.2840	0.0341*	
H10	0.7797	−0.0232	0.1812	0.0320*	
H11	0.5042	0.0144	0.0961	0.0295*	
H14	0.0347	0.0830	0.1011	0.0277*	
H16	−0.3098	0.2704	0.0726	0.0264*	
H17	−0.2766	0.1891	−0.0062	0.0291*	
H18	−0.0363	0.1794	−0.1121	0.0312*	
H19	0.1825	0.2498	−0.1417	0.0295*	
H22	0.3787	0.3520	−0.1516	0.0325*	
H23	0.4702	0.4425	−0.1371	0.0365*	
H24	0.3238	0.4978	−0.0327	0.0338*	
H25	0.0795	0.4640	0.0597	0.0276*	
H28	−0.4362	0.3885	0.0566	0.0295*	

### Atomic displacement parameters (Å^2^)

**Table:**

	*U*^11^	*U*^22^	*U*^33^	*U*^12^	*U*^13^	*U*^23^
Cl1	0.02329 (18)	0.0368 (2)	0.02081 (18)	−0.00867 (14)	0.00395 (14)	−0.00355 (14)
Cl2	0.02099 (17)	0.02764 (19)	0.0290 (2)	−0.00531 (13)	−0.00099 (14)	0.00015 (14)
Cl3	0.0287 (2)	0.0402 (3)	0.0383 (3)	0.00770 (16)	0.01367 (17)	−0.00693 (17)
Cl4	0.0270 (2)	0.0474 (3)	0.02074 (19)	0.00283 (16)	−0.00039 (14)	−0.00556 (15)
N1	0.0252 (7)	0.0203 (6)	0.0198 (6)	0.0011 (5)	−0.0025 (5)	−0.0015 (5)
N2	0.0192 (6)	0.0178 (6)	0.0196 (6)	−0.0010 (5)	0.0057 (5)	−0.0012 (5)
C1	0.0248 (7)	0.0215 (7)	0.0160 (7)	−0.0014 (6)	0.0019 (6)	−0.0020 (5)
C2	0.0239 (7)	0.0259 (8)	0.0211 (7)	0.0025 (6)	−0.0001 (6)	−0.0015 (6)
C3	0.0283 (8)	0.0239 (8)	0.0249 (8)	0.0028 (6)	0.0056 (6)	−0.0018 (6)
C4	0.0326 (8)	0.0246 (8)	0.0222 (8)	−0.0035 (6)	0.0052 (6)	−0.0055 (6)
C5	0.0270 (8)	0.0271 (8)	0.0178 (7)	−0.0031 (6)	0.0009 (6)	−0.0026 (6)
C6	0.0232 (7)	0.0242 (7)	0.0155 (7)	−0.0007 (6)	0.0019 (6)	0.0006 (5)
C7	0.0244 (7)	0.0240 (7)	0.0166 (7)	−0.0003 (6)	0.0027 (6)	0.0008 (6)
C8	0.0268 (8)	0.0333 (8)	0.0189 (7)	0.0004 (7)	−0.0010 (6)	0.0018 (6)
C9	0.0275 (8)	0.0336 (9)	0.0241 (8)	0.0049 (7)	0.0015 (6)	0.0083 (7)
C10	0.0311 (8)	0.0233 (7)	0.0264 (8)	0.0039 (6)	0.0065 (7)	0.0061 (6)
C11	0.0291 (8)	0.0226 (7)	0.0221 (8)	−0.0001 (6)	0.0025 (6)	0.0017 (6)
C12	0.0238 (7)	0.0229 (7)	0.0173 (7)	0.0002 (6)	0.0016 (6)	0.0033 (6)
C13	0.0236 (7)	0.0190 (7)	0.0189 (7)	0.0001 (6)	0.0016 (6)	−0.0015 (5)
C14	0.0246 (8)	0.0236 (7)	0.0212 (7)	−0.0016 (6)	0.0020 (6)	0.0012 (6)
C15	0.0200 (7)	0.0191 (7)	0.0161 (7)	0.0023 (5)	0.0003 (5)	−0.0013 (5)
C16	0.0222 (7)	0.0223 (7)	0.0216 (7)	−0.0014 (6)	0.0024 (6)	0.0006 (6)
C17	0.0285 (8)	0.0191 (7)	0.0246 (8)	−0.0019 (6)	−0.0033 (6)	0.0005 (6)
C18	0.0347 (9)	0.0215 (7)	0.0210 (8)	0.0050 (6)	−0.0039 (6)	−0.0043 (6)
C19	0.0290 (8)	0.0271 (8)	0.0179 (7)	0.0058 (6)	0.0028 (6)	−0.0028 (6)
C20	0.0217 (7)	0.0228 (7)	0.0162 (7)	0.0027 (6)	0.0016 (6)	0.0010 (5)
C21	0.0202 (7)	0.0243 (7)	0.0187 (7)	0.0021 (6)	0.0027 (6)	0.0014 (6)
C22	0.0236 (8)	0.0345 (9)	0.0238 (8)	0.0008 (6)	0.0073 (6)	0.0024 (6)
C23	0.0232 (8)	0.0380 (9)	0.0310 (9)	−0.0032 (7)	0.0078 (7)	0.0097 (7)
C24	0.0233 (8)	0.0254 (8)	0.0359 (9)	−0.0034 (6)	0.0017 (7)	0.0067 (7)
C25	0.0216 (7)	0.0210 (7)	0.0265 (8)	0.0014 (6)	0.0025 (6)	0.0016 (6)
C26	0.0174 (7)	0.0217 (7)	0.0189 (7)	0.0008 (5)	0.0031 (6)	0.0031 (5)
C27	0.0215 (7)	0.0173 (6)	0.0178 (7)	−0.0000 (5)	0.0037 (6)	−0.0006 (5)
C28	0.0229 (7)	0.0264 (8)	0.0249 (8)	0.0025 (6)	0.0047 (6)	−0.0028 (6)

### Geometric parameters (Å, º)

**Table:**

Cl1—C13	1.7470 (16)	C18—C19	1.385 (3)
Cl2—C14	1.7162 (16)	C19—C20	1.399 (2)
Cl3—C28	1.7146 (17)	C20—C21	1.452 (2)
Cl4—C27	1.7448 (14)	C21—C22	1.400 (3)
N1—C1	1.405 (2)	C21—C26	1.406 (3)
N1—C12	1.4051 (19)	C22—C23	1.384 (3)
N1—C13	1.4028 (19)	C23—C24	1.401 (3)
N2—C15	1.405 (2)	C24—C25	1.390 (3)
N2—C26	1.401 (2)	C25—C26	1.388 (2)
N2—C27	1.3967 (19)	C27—C28	1.318 (2)
C1—C2	1.390 (3)	C2—H2	0.899
C1—C6	1.404 (2)	C3—H3	0.979
C2—C3	1.391 (2)	C4—H4	0.965
C3—C4	1.404 (3)	C5—H5	0.956
C4—C5	1.386 (3)	C8—H8	0.950
C5—C6	1.396 (2)	C9—H9	0.950
C6—C7	1.448 (3)	C10—H10	0.950
C7—C8	1.397 (3)	C11—H11	0.950
C7—C12	1.409 (2)	C14—H14	0.950
C8—C9	1.388 (3)	C16—H16	0.950
C9—C10	1.398 (3)	C17—H17	0.950
C10—C11	1.393 (3)	C18—H18	0.950
C11—C12	1.390 (3)	C19—H19	0.950
C13—C14	1.322 (3)	C22—H22	0.950
C15—C16	1.389 (2)	C23—H23	0.950
C15—C20	1.409 (2)	C24—H24	0.950
C16—C17	1.392 (3)	C25—H25	0.950
C17—C18	1.399 (3)	C28—H28	0.950
			
Cl1···Cl2	3.2228 (6)	C18···H5^v^	2.7316
Cl1···C1	3.4920 (15)	C19···H2	2.9494
Cl1···C12	3.4398 (16)	C19···H5^v^	2.7428
Cl3···Cl4	3.1799 (7)	C20···H2	3.0357
Cl4···C25	3.5615 (16)	C20···H3	3.3426
Cl4···C26	3.3171 (16)	C20···H5^v^	3.0889
N1···C5	3.597 (2)	C20···H8^v^	3.0187
N1···C8	3.600 (2)	C21···H3	3.5416
N2···C19	3.599 (2)	C21···H8^v^	2.7081
N2···C22	3.597 (2)	C21···H28^viii^	3.1440
C1···C4	2.754 (2)	C22···H8^v^	2.9408
C1···C14	3.373 (2)	C22···H28^viii^	2.8988
C2···C5	2.838 (3)	C23···H8^v^	3.5261
C2···C13	3.103 (3)	C23···H9^v^	3.2336
C3···C6	2.776 (3)	C23···H24^vii^	3.3031
C5···C8	3.364 (3)	C23···H28^viii^	2.7690
C7···C10	2.777 (3)	C24···H9^v^	3.2476
C8···C11	2.840 (3)	C24···H24^vii^	2.9362
C9···C12	2.757 (3)	C24···H25^ix^	3.0478
C11···C13	3.086 (2)	C24···H28^viii^	2.8965
C12···C14	3.383 (2)	C25···H24^ix^	3.4426
C15···C18	2.750 (2)	C25···H25^ix^	2.9418
C15···C28	3.268 (3)	C25···H28^viii^	3.1508
C16···C19	2.845 (3)	C26···H3	3.3872
C16···C27	3.121 (2)	C26···H8^v^	3.1601
C16···C28	3.552 (3)	C26···H28^viii^	3.2532
C17···C20	2.777 (3)	C27···H3	3.5100
C19···C22	3.380 (3)	C27···H24^ix^	3.4402
C21···C24	2.779 (3)	C28···H3^x^	3.5917
C22···C25	2.835 (3)	C28···H4^x^	3.3679
C23···C26	2.753 (3)	C28···H24^ix^	2.9922
C25···C27	3.058 (2)	H2···C15	3.0145
C26···C28	3.455 (3)	H2···C16	2.9458
Cl3···C10^i^	3.4335 (17)	H2···C17	2.8276
C5···C18^ii^	3.561 (3)	H2···C18	2.8363
C6···C22^iii^	3.544 (3)	H2···C19	2.9494
C10···Cl3^iv^	3.4335 (17)	H2···C20	3.0357
C18···C5^v^	3.561 (3)	H2···H16	3.5041
C22···C6^vi^	3.544 (3)	H2···H17	3.3319
C24···C24^vii^	3.590 (3)	H2···H18	3.3495
C24···C28^viii^	3.577 (3)	H2···H19	3.5045
C25···C25^ix^	3.447 (3)	H3···Cl3^viii^	3.4784
C28···C24^x^	3.577 (3)	H3···Cl4	3.0416
Cl1···H11	3.5722	H3···N2	3.0933
Cl1···H14	3.5605	H3···C15	3.0613
Cl4···H25	3.1879	H3···C20	3.3426
Cl4···H28	3.5485	H3···C21	3.5416
N1···H2	2.7730	H3···C26	3.3872
N1···H11	2.7865	H3···C27	3.5100
N1···H14	2.5278	H3···C28^viii^	3.5917
N2···H16	2.7844	H3···H16^viii^	3.3353
N2···H25	2.7733	H3···H19^iii^	3.2182
N2···H28	2.5438	H3···H28^viii^	3.4987
C1···H3	3.2509	H4···Cl1^iii^	3.4214
C1···H5	3.2864	H4···Cl3^viii^	3.0689
C1···H14	3.4021	H4···C17^ii^	3.5888
C2···H4	3.2978	H4···C18^ii^	3.4790
C3···H5	3.2923	H4···C28^viii^	3.3679
C4···H2	3.2460	H4···H16^viii^	3.2639
C5···H3	3.3121	H4···H17^ii^	3.4969
C5···H8	3.2340	H4···H18^ii^	3.3021
C6···H2	3.2674	H4···H19^iii^	3.0240
C6···H4	3.2685	H5···C15^ii^	3.4029
C6···H8	2.8858	H5···C16^ii^	3.4385
C7···H5	2.8814	H5···C17^ii^	3.0959
C7···H9	3.2564	H5···C18^ii^	2.7316
C7···H11	3.3123	H5···C19^ii^	2.7428
C8···H5	3.2289	H5···C20^ii^	3.0889
C8···H10	3.2721	H5···H16^viii^	3.5424
C9···H11	3.2980	H5···H18^ii^	3.0896
C10···H8	3.2826	H5···H19^ii^	3.1165
C11···H9	3.2855	H5···H22^iii^	3.1651
C12···H8	3.2874	H8···C15^ii^	3.5732
C12···H10	3.2344	H8···C20^ii^	3.0187
C12···H14	3.4323	H8···C21^ii^	2.7081
C13···H2	2.9366	H8···C22^ii^	2.9408
C13···H11	2.8985	H8···C23^ii^	3.5261
C14···H2	3.3756	H8···C26^ii^	3.1601
C14···H11	3.3896	H8···H14^viii^	3.2952
C15···H17	3.2313	H8···H22^ii^	3.2363
C15···H19	3.2881	H8···H23^iii^	3.4728
C15···H28	3.2084	H9···Cl4^xiv^	3.5625
C16···H18	3.2837	H9···C23^ii^	3.2336
C16···H28	3.4228	H9···C24^ii^	3.2476
C17···H19	3.2845	H9···H14^viii^	3.0855
C18···H16	3.2985	H9···H23^ii^	3.4952
C19···H17	3.2728	H9···H24^ii^	3.5198
C19···H22	3.2549	H9···H25^xiv^	2.8658
C20···H16	3.3147	H10···Cl1^xi^	2.9660
C20···H18	3.2517	H10···Cl2^xi^	3.0910
C20···H22	2.8968	H10···Cl3^iv^	3.0713
C21···H19	2.8921	H10···Cl4^xiv^	3.0947
C21···H23	3.2563	H10···H14^viii^	3.3971
C21···H25	3.3081	H11···Cl1^xi^	3.3608
C22···H19	3.2547	H11···Cl2^xii^	3.5410
C22···H24	3.2720	H11···H11^xi^	2.9440
C23···H25	3.2949	H14···C7^x^	3.1972
C24···H22	3.2820	H14···C8^x^	2.8595
C25···H23	3.2821	H14···C9^x^	2.7134
C26···H22	3.2833	H14···C10^x^	2.9352
C26···H24	3.2335	H14···C11^x^	3.2952
C26···H28	3.5745	H14···C12^x^	3.3900
C27···H16	2.9378	H14···H8^x^	3.2952
C27···H25	2.8576	H14···H9^x^	3.0855
C28···H16	3.1280	H14···H10^x^	3.3971
C28···H25	3.5708	H16···C1^x^	3.2646
H2···H3	2.3023	H16···C2^x^	3.1159
H2···H14	3.4913	H16···C3^x^	2.8545
H3···H4	2.3793	H16···C4^x^	2.8063
H4···H5	2.3554	H16···C5^x^	3.0031
H5···H8	2.8345	H16···C6^x^	3.2160
H8···H9	2.3412	H16···H2	3.5041
H9···H10	2.3307	H16···H3^x^	3.3353
H10···H11	2.3524	H16···H4^x^	3.2639
H11···H14	3.5650	H16···H5^x^	3.5424
H16···H17	2.3541	H17···Cl1^x^	3.0834
H16···H28	3.0605	H17···Cl2	3.3739
H17···H18	2.3293	H17···N1^x^	3.3794
H18···H19	2.3371	H17···C1^x^	3.1899
H19···H22	2.8670	H17···C6^x^	3.3969
H22···H23	2.3361	H17···C14	3.5290
H23···H24	2.3344	H17···H2	3.3319
H24···H25	2.3504	H17···H4^v^	3.4969
Cl1···H4^vi^	3.4214	H18···Cl1	3.1696
Cl1···H10^xi^	2.9660	H18···Cl2	2.9554
Cl1···H11^xi^	3.3608	H18···Cl4^vi^	2.9791
Cl1···H17^viii^	3.0834	H18···C13	3.3975
Cl1···H18	3.1696	H18···C14	3.3118
Cl2···H10^xi^	3.0910	H18···H2	3.3495
Cl2···H11^xii^	3.5410	H18···H4^v^	3.3021
Cl2···H17	3.3739	H18···H5^v^	3.0896
Cl2···H18	2.9554	H19···C3^vi^	3.0321
Cl3···H3^x^	3.4784	H19···C4^vi^	2.9258
Cl3···H4^x^	3.0689	H19···C5^vi^	3.4787
Cl3···H10^i^	3.0713	H19···H2	3.5045
Cl3···H24^ix^	3.5117	H19···H3^vi^	3.2182
Cl4···H3	3.0416	H19···H4^vi^	3.0240
Cl4···H9^xiii^	3.5625	H19···H5^v^	3.1165
Cl4···H10^xiii^	3.0947	H22···N1^vi^	3.5838
Cl4···H18^iii^	2.9791	H22···C1^vi^	3.1079
N1···H17^viii^	3.3794	H22···C4^vi^	3.5404
N1···H22^iii^	3.5838	H22···C5^vi^	2.9023
N2···H3	3.0933	H22···C6^vi^	2.6407
C1···H16^viii^	3.2646	H22···C7^vi^	2.9498
C1···H17^viii^	3.1899	H22···C8^vi^	3.5422
C1···H22^iii^	3.1079	H22···C12^vi^	3.5224
C2···H16^viii^	3.1159	H22···H5^vi^	3.1651
C3···H16^viii^	2.8545	H22···H8^v^	3.2363
C3···H19^iii^	3.0321	H22···H28^viii^	3.3783
C4···H16^viii^	2.8063	H23···C7^vi^	3.1181
C4···H19^iii^	2.9258	H23···C8^vi^	3.0725
C4···H22^iii^	3.5404	H23···C9^vi^	3.2355
C5···H16^viii^	3.0031	H23···C10^vi^	3.4587
C5···H19^iii^	3.4787	H23···C11^vi^	3.5249
C5···H22^iii^	2.9023	H23···C12^vi^	3.3288
C6···H16^viii^	3.2160	H23···H8^vi^	3.4728
C6···H17^viii^	3.3969	H23···H9^v^	3.4952
C6···H22^iii^	2.6407	H23···H24^vii^	3.1628
C7···H14^viii^	3.1972	H23···H28^viii^	3.2006
C7···H22^iii^	2.9498	H24···Cl3^ix^	3.5117
C7···H23^iii^	3.1181	H24···C23^vii^	3.3031
C8···H14^viii^	2.8595	H24···C24^vii^	2.9362
C8···H22^iii^	3.5422	H24···C25^ix^	3.4426
C8···H23^iii^	3.0725	H24···C27^ix^	3.4402
C9···H14^viii^	2.7134	H24···C28^ix^	2.9922
C9···H23^iii^	3.2355	H24···H9^v^	3.5198
C9···H25^xiv^	3.4703	H24···H23^vii^	3.1628
C10···H14^viii^	2.9352	H24···H24^vii^	2.4817
C10···H23^iii^	3.4587	H24···H25^ix^	2.8508
C11···H14^viii^	3.2952	H24···H28^viii^	3.3800
C11···H23^iii^	3.5249	H24···H28^ix^	2.9544
C12···H14^viii^	3.3900	H25···C9^xiii^	3.4703
C12···H22^iii^	3.5224	H25···C24^ix^	3.0478
C12···H23^iii^	3.3288	H25···C25^ix^	2.9418
C13···H18	3.3975	H25···H9^xiii^	2.8658
C14···H17	3.5290	H25···H24^ix^	2.8508
C14···H18	3.3118	H25···H25^ix^	2.6885
C15···H2	3.0145	H28···C21^x^	3.1440
C15···H3	3.0613	H28···C22^x^	2.8988
C15···H5^v^	3.4029	H28···C23^x^	2.7690
C15···H8^v^	3.5732	H28···C24^x^	2.8965
C16···H2	2.9458	H28···C25^x^	3.1508
C16···H5^v^	3.4385	H28···C26^x^	3.2532
C17···H2	2.8276	H28···H3^x^	3.4987
C17···H4^v^	3.5888	H28···H22^x^	3.3783
C17···H5^v^	3.0959	H28···H23^x^	3.2006
C18···H2	2.8363	H28···H24^x^	3.3800
C18···H4^v^	3.4790	H28···H24^ix^	2.9544
			
C1—N1—C12	108.34 (12)	C23—C24—C25	121.32 (15)
C1—N1—C13	125.34 (12)	C24—C25—C26	117.21 (14)
C12—N1—C13	124.16 (13)	N2—C26—C21	108.84 (13)
C15—N2—C26	108.57 (12)	N2—C26—C25	128.60 (14)
C15—N2—C27	126.75 (12)	C21—C26—C25	122.48 (14)
C26—N2—C27	123.95 (12)	Cl4—C27—N2	115.76 (11)
N1—C1—C2	128.71 (13)	Cl4—C27—C28	121.61 (12)
N1—C1—C6	108.77 (13)	N2—C27—C28	122.63 (13)
C2—C1—C6	122.48 (14)	Cl3—C28—C27	123.51 (13)
C1—C2—C3	117.01 (14)	C1—C2—H2	122.524
C2—C3—C4	121.42 (14)	C3—C2—H2	120.422
C3—C4—C5	120.80 (14)	C2—C3—H3	117.652
C4—C5—C6	118.75 (14)	C4—C3—H3	120.905
C1—C6—C5	119.52 (14)	C3—C4—H4	119.329
C1—C6—C7	107.20 (13)	C5—C4—H4	119.852
C5—C6—C7	133.22 (14)	C4—C5—H5	120.759
C6—C7—C8	133.40 (14)	C6—C5—H5	120.472
C6—C7—C12	107.14 (13)	C7—C8—H8	120.630
C8—C7—C12	119.44 (14)	C9—C8—H8	120.632
C7—C8—C9	118.74 (15)	C8—C9—H9	119.584
C8—C9—C10	120.85 (15)	C10—C9—H9	119.567
C9—C10—C11	121.59 (15)	C9—C10—H10	119.207
C10—C11—C12	117.03 (14)	C11—C10—H10	119.200
N1—C12—C7	108.52 (13)	C10—C11—H11	121.473
N1—C12—C11	129.10 (14)	C12—C11—H11	121.492
C7—C12—C11	122.34 (14)	Cl2—C14—H14	117.993
Cl1—C13—N1	115.89 (11)	C13—C14—H14	117.994
Cl1—C13—C14	122.55 (12)	C15—C16—H16	121.610
N1—C13—C14	121.56 (14)	C17—C16—H16	121.621
Cl2—C14—C13	124.01 (13)	C16—C17—H17	119.231
N2—C15—C16	128.92 (14)	C18—C17—H17	119.224
N2—C15—C20	108.42 (13)	C17—C18—H18	119.401
C16—C15—C20	122.64 (14)	C19—C18—H18	119.399
C15—C16—C17	116.77 (14)	C18—C19—H19	120.775
C16—C17—C18	121.54 (15)	C20—C19—H19	120.755
C17—C18—C19	121.20 (14)	C21—C22—H22	120.587
C18—C19—C20	118.47 (15)	C23—C22—H22	120.576
C15—C20—C19	119.38 (14)	C22—C23—H23	119.542
C15—C20—C21	107.22 (13)	C24—C23—H23	119.540
C19—C20—C21	133.39 (14)	C23—C24—H24	119.338
C20—C21—C22	133.71 (15)	C25—C24—H24	119.337
C20—C21—C26	106.94 (13)	C24—C25—H25	121.396
C22—C21—C26	119.20 (14)	C26—C25—H25	121.394
C21—C22—C23	118.84 (16)	Cl3—C28—H28	118.251
C22—C23—C24	120.92 (16)	C27—C28—H28	118.239
			
C1—N1—C12—C7	2.05 (16)	C6—C7—C12—N1	−1.31 (16)
C1—N1—C12—C11	179.83 (13)	C6—C7—C12—C11	−179.27 (12)
C12—N1—C1—C2	−179.56 (13)	C8—C7—C12—N1	177.13 (13)
C12—N1—C1—C6	−2.00 (15)	C8—C7—C12—C11	−0.8 (3)
C1—N1—C13—Cl1	82.69 (17)	C12—C7—C8—C9	0.2 (3)
C1—N1—C13—C14	−97.54 (17)	C7—C8—C9—C10	0.6 (3)
C13—N1—C1—C2	16.6 (3)	C8—C9—C10—C11	−0.8 (3)
C13—N1—C1—C6	−165.81 (12)	C9—C10—C11—C12	0.2 (3)
C12—N1—C13—Cl1	−78.65 (17)	C10—C11—C12—N1	−176.90 (14)
C12—N1—C13—C14	101.12 (17)	C10—C11—C12—C7	0.6 (3)
C13—N1—C12—C7	166.09 (12)	Cl1—C13—C14—Cl2	−0.3 (2)
C13—N1—C12—C11	−16.1 (3)	N1—C13—C14—Cl2	179.91 (11)
C15—N2—C26—C21	0.20 (15)	N2—C15—C16—C17	178.30 (12)
C15—N2—C26—C25	177.01 (12)	N2—C15—C20—C19	−178.78 (11)
C26—N2—C15—C16	−178.37 (12)	N2—C15—C20—C21	−0.11 (14)
C26—N2—C15—C20	−0.06 (14)	C16—C15—C20—C19	−0.3 (2)
C15—N2—C27—Cl4	102.36 (14)	C16—C15—C20—C21	178.33 (12)
C15—N2—C27—C28	−78.22 (17)	C20—C15—C16—C17	0.2 (2)
C27—N2—C15—C16	11.2 (3)	C15—C16—C17—C18	−0.2 (2)
C27—N2—C15—C20	−170.47 (12)	C16—C17—C18—C19	0.4 (3)
C26—N2—C27—Cl4	−66.67 (16)	C17—C18—C19—C20	−0.6 (3)
C26—N2—C27—C28	112.75 (15)	C18—C19—C20—C15	0.5 (2)
C27—N2—C26—C21	170.95 (11)	C18—C19—C20—C21	−177.74 (13)
C27—N2—C26—C25	−12.2 (3)	C15—C20—C21—C22	−175.29 (13)
N1—C1—C2—C3	176.52 (13)	C15—C20—C21—C26	0.23 (15)
N1—C1—C6—C5	−176.32 (11)	C19—C20—C21—C22	3.1 (3)
N1—C1—C6—C7	1.17 (15)	C19—C20—C21—C26	178.64 (15)
C2—C1—C6—C5	1.4 (3)	C20—C21—C22—C23	174.66 (14)
C2—C1—C6—C7	178.91 (13)	C20—C21—C26—N2	−0.26 (15)
C6—C1—C2—C3	−0.7 (3)	C20—C21—C26—C25	−177.31 (12)
C1—C2—C3—C4	−0.4 (3)	C22—C21—C26—N2	176.03 (12)
C2—C3—C4—C5	0.9 (3)	C22—C21—C26—C25	−1.0 (2)
C3—C4—C5—C6	−0.2 (3)	C26—C21—C22—C23	−0.4 (2)
C4—C5—C6—C1	−0.9 (3)	C21—C22—C23—C24	1.1 (3)
C4—C5—C6—C7	−177.62 (14)	C22—C23—C24—C25	−0.3 (3)
C1—C6—C7—C8	−178.05 (15)	C23—C24—C25—C26	−1.1 (3)
C1—C6—C7—C12	0.09 (16)	C24—C25—C26—N2	−174.65 (13)
C5—C6—C7—C8	−1.0 (3)	C24—C25—C26—C21	1.8 (2)
C5—C6—C7—C12	177.09 (15)	Cl4—C27—C28—Cl3	0.12 (19)
C6—C7—C8—C9	178.15 (15)	N2—C27—C28—Cl3	−179.27 (11)

Symmetry codes: (i) −*x*, *y*+1/2, −*z*+1/2; (ii) *x*+1, −*y*+1/2, *z*+1/2; (iii) *x*, −*y*+1/2, *z*+1/2; (iv) −*x*, *y*−1/2, −*z*+1/2; (v) *x*−1, −*y*+1/2, *z*−1/2; (vi) *x*, −*y*+1/2, *z*−1/2; (vii) −*x*+1, −*y*+1, −*z*; (viii) *x*+1, *y*, *z*; (ix) −*x*, −*y*+1, −*z*; (x) *x*−1, *y*, *z*; (xi) −*x*+1, −*y*, −*z*; (xii) −*x*, −*y*, −*z*; (xiii) −*x*+1, *y*+1/2, −*z*+1/2; (xiv) −*x*+1, *y*−1/2, −*z*+1/2.
